# A 4 mm-Long Implant Rehabilitation in the Posterior Maxilla with Dynamic Navigation Technology: A Case Report after a Three-Years Post-Loading Follow-Up

**DOI:** 10.3390/ijerph18189808

**Published:** 2021-09-17

**Authors:** Gerardo Pellegrino, Giuseppe Lizio, Fabio Rossi, Lorenzo Tuci, Lorenzo Ferraioli, Luigi Vito Stefanelli, Stefano Di Carlo, Francesca De Angelis

**Affiliations:** 1 Oral Surgery Unit, Department of Biomedical and Neuromotor Sciences, University of Bologna, Via San Vitale 59, 40125 Bologna, Italy; gerardo.pellegrino2@unibo.it (G.P.); fabio.rossi33@unibo.it (F.R.); lorenzotuci90@gmail.com (L.T.); lorenzoferraioli@gmail.com (L.F.); 2 Department of Oral and Maxillo-Facial Sciences, Sapienza University of Rome, 00185 Rome, Italy; gigistef@libero.it (L.V.S.); stefano.dicarlo@uniroma1.it (S.D.C.); francesca.deangelis@uniroma1.it (F.D.A.)

**Keywords:** ultra-short implants, computer-aided implantology, dynamic navigation

## Abstract

The use of short (<8 mm long) and ultra-short (<6 mm long) implants allows the prosthetic rehabilitation of the posterior ridges of the jaws avoiding reconstructive procedures. Nevertheless, this approach requires vast experience to ensure the primary stability of the fixture in a correct position. Computer-aided implantology (CAI) achieves better results than the free-hand one in terms of placement accuracy, reducing the surgical risks and the operative timings. Dynamic navigation (DN) allows the surgeon to track the position and movements of the drill in real-time on the CT imaging data set. It is more versatile than the computed static system, enabling the operator to change the guidance coordinates according to the intra-operative feedbacks. A mono-edentulous upper right first molar site was rehabilitated with a four mm-long implant to avoid reconstructive techniques, drastically rejected by the patients. The case was managed within a DN protocol considering the minimal available bone and the prosthetic demands. The phases of this procedure were strictly documented up to a 3-year follow-up. No intra-operative problems occurred, and adequate primary stability of the implant was obtained. The prosthetic loading was carried out within only six weeks without any complications. No variation of the baseline clinical scenario as evidenced clinically and radiographically at the end of follow-up. No similar cases are reported in the literature.

## 1. Introduction

In vitro studies indicate that functional loads are primarily concentrated in the crestal region of dental implants [1,2,3]. Since then, the rule of a crown-to-implant ratio (C/I) ≤1 was discussed, and the use of short dental implants emerged. This approach has expanded, with a progressive reduction of the fixtures’ length for managing more and more vertically impaired alveolar sites. Hence, the definition of “short implant” changed from ≤10 mm to 6 mm [4,5] up to the “ultra-short” measure of 4 mm in length. Systematic reviews on ≤6 mm-short implants report a range of 90–98.5% of the mean survival rate values in posterior maxilla up to 5 years [5,6,7,8,9]. Regarding 4 mm short implants, only two randomized controlled trials (RCTs), both in the mandible, report good outcomes in atrophic bone, compared to reconstructed sites with one-year follow-up [10,11].

A recent study reported how the prosthetic superstructure height and the C/I ratio do not influence the results up to a 3/1 value at least for ≥5 mm long screws, independently from a single unit or splinted prosthetic solutions [12]. Lombardo et al. recorded a cumulative success rate of 97.1 % for 6/5 long implants in posterior jaws, loaded with single crowns after three years of follow-up [13]. Up to a 9-year follow-up, similar clinical outcomes were observed in single and splinted porous-surfaced implants shorter than 10 mm in association with a sinus lift [14]. Minimal peri-implant bone resorption was reported on 5-to-8 mm length fixtures associated with trans-crestal sinus lift after three years of loading with a single unit prosthesis [15]. After five years of loading, a mean marginal bone loss of 0.38 and 0.48 was reported in the C/I < 2 and C/I ≥ 2 groups, respectively. The linear regression model failed to find a correlation between the C/I ratio and marginal bone loss over time [16]. The survival rate of 4-mm long screws in 18 posterior mandibles reached 100% at three years, lowering to 91.7% with 6 mm ones in both jaws at ten years- loading [5]. No single crown prosthetic rehabilitation was reported using 4-mm long implants. In particular, great importance was attributed to avoid oblique direction loading on ultra-short screws implants [17]. Since the attainment of primary stability and prosthetically correct positioning condition the osseointegration and the survival of short implants, the surgical site preparation and the surface properties and the design of the fixtures have great importance [7]. In the dynamic navigation (DN) system, one or two cameras detect in real-time the position of reference tools placed on the patient and the surgical handle-piece, and software pairs this information with the CT images data. This technology allows the real-time tracking on a screen of the surgical tool’s position onto the patient’s 3D radiological imaging, with a superimposition of the instrument’s tip on the planned position graphics [18,19]. This technology has been simplified with less bulky instrumentation and has become more and more diffused. The digital-imaging guidance works, letting the operator maintain the complete intra-operatory sensorial feedback, modifying the site preparation modality, and even changing the virtual project in case of problems [18,19,20,21]. The reliability in implant positioning accuracy using standard or tilted implants have been widely demonstrated to be superior with the DN support than without, with about 1 mm of the discrepancy between planned and actual positions [18,21,22]. The more complicated the situation, the more useful this approach is, reducing the learning curve to allow treatment of even complex cases after a limited training period. The posterior atrophic maxilla rehabilitation with short-implants is complicated by its soft cancellous bone structure and considerable masticatory forces [23], especially in this reported mono-edentulism case, with only a 4 mm-long implant to be used supporting a high single-crown prosthesis. Hence, to limit the risk of pre-and post-loading failure, dynamic navigation (DN) support to implant placement procedure is adopted to accurately transfer in the clinic reality the implant position, digitally planned according to the anatomical and prosthetic requirements. Since no similar cases are documented in the literature so far, it is decided to reveal it to a broader audience considering the good results after three years of loading.

## 2. Materials and Methods

### 2.1. Planning

**Figure 1:**
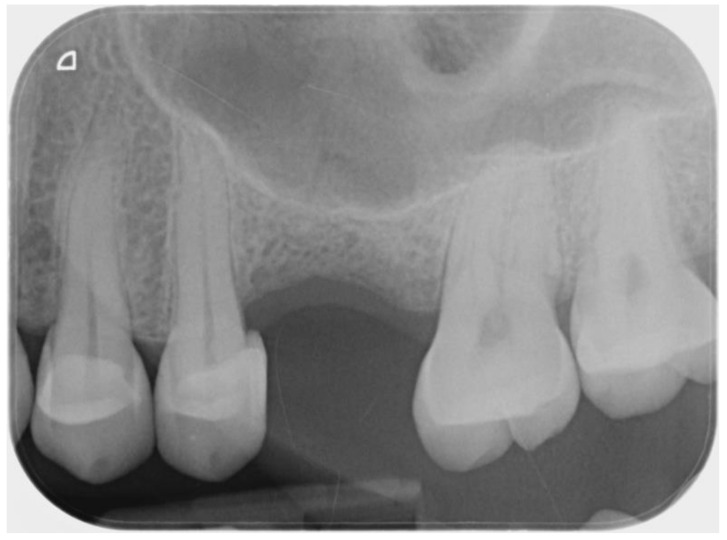
Intra-oral pre-operative x-ray of the edentulous site.

A female patient, forty-seven years old, required the rehabilitation of an atrophic mono-edentulous zone corresponding to the upper right first molar. Periapical x-rays and cone beam computed tomography confirmed severe bone resorption resulting in a residual bone height of ≤5 mm (Figure 1). The patient expressly required a mini-invasive treatment to be performed in one surgical setting and rejected any kind of grafting procedure. Furthermore, she did not accept the fixed bridge solution, not intending to involve the nearby teeth. The option of ultra-short 4 mm-long and 4.1-mm wide implant-borne rehabilitation was eventually proposed. The patient was informed about the limited follow-up, five years, of this type of rehabilitation and the poor survival rate reported in the literature, particularly in the maxilla. As regards prosthetic finalization, the need for a high crown with poor esthetic results was also underlined. In order to favor the obtainment of sufficient primary stability, sparing the residual bone as much as possible, with a prosthetically correct sitting of the screw, the usefulness of DN technology support was expressed to the patient. She signed a precisely informed consent summing up what was aforementioned.

The ImplaNav dynamic navigation system (BresMedical, Sydney, Australia) was employed. First, a pre-operative CBCT scan was taken with the markers plate (MP) anchored to the patient’s residual teeth with a tray-like tool filled with a high-density impression material (Ramitec, 3M ESPE, St Paul, MN, USA). The anchor impression involved three healthy teeth of the upper jaw. In the case of total edentulism, not reported in this paper, a narrow-implant, flapless inserted at the maxilla’s median point, works as anchorage. After taking the CBCT, the reference system was removed and stored to be positioned in situ during the surgical procedure.

The MP carries the fiducial markers. These serve for the registration process at the time of the surgery. The Digital Imaging and Communication in Medicine (DICOM) data, related to the features of the hard tissue, and the 3D standard tessellation (STL) data, related to the teeth and soft tissues surfaces, from the scanning of the waxed-up models, were paired by a dedicated software to choose the implant characteristics and positions according to the anatomical and prosthetic demands. The virtual positioned implant will be graphically evident during surgery.

At surgery time, a registration/calibration process must be accomplished to allow the software to match the pre-operative CBCT reporting the virtual implant in position with the surgical tool while operating in the patient’s mouth. MP with the fiducial markers is replaced at the CBCT time, enabling the system to pair the fiducial markers in the CBCT data with the ones attached to the patient. Furthermore, the MP has attached the patient reference tool (pRF), while a second reference tool is attached to the handpiece (HRT). Both these RFs consisted of support for three reflective spheres with different geometries. An infrared camera (NDI Polaris Vicra; Northern Digital, Waterloo, ON, Canada) detects the spatial position of the RFs, contemporarily coupled with each other. This can record both the patient’s and the surgical handpiece’s position, and the system could display the drill position on the patient’s CBCT images. To carry out the registration/calibration process, the operator exposes the hRT attached to the handpiece for at least three seconds to the camera that can now match it with the pRT. After touching the fiducial markers on the MP in sequence with a lancet drill (calibration tool) whose length is known to the software, it is unnecessary to repeat the calibration for the following drills already stored in the digital library. The software can now superimpose the surgical tool position and axis to the 3D radiological imaging corresponding to the patient’s surgical site, acquired pre-operatively [18,21,22].

### 2.2. Surgical and Prosthetic Procedure

**Figure 2:**
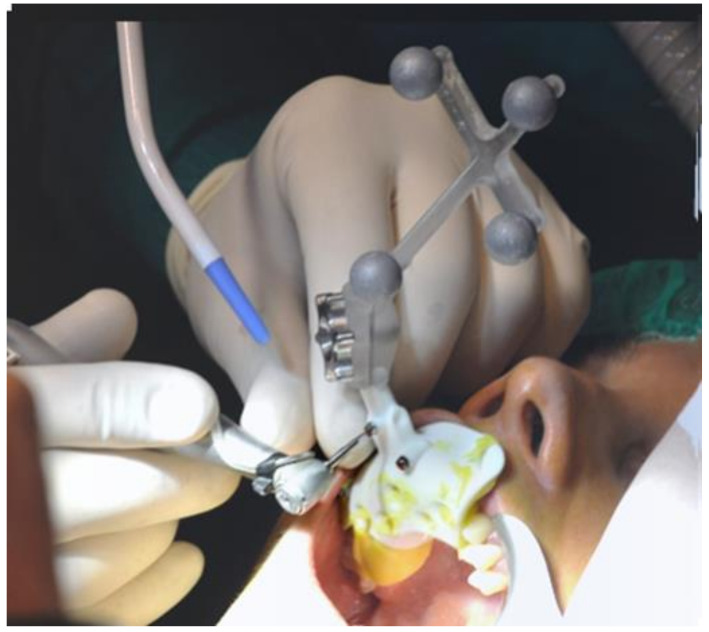
The registration/calibration just before the surgical procedure.

**Figure 3:**
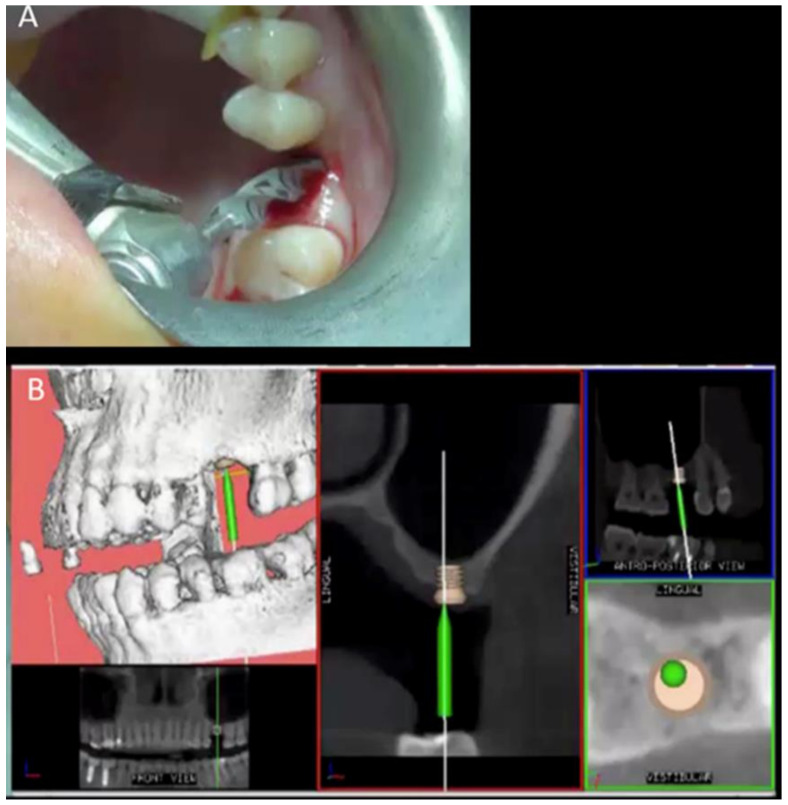
Clinical view of the implant site preparation (**A**) and the screen view showing the final implant digital position, and the green pin representing the working burr superimposed on the 3D radiologic anatomy (**B**).

**Figure 4:**
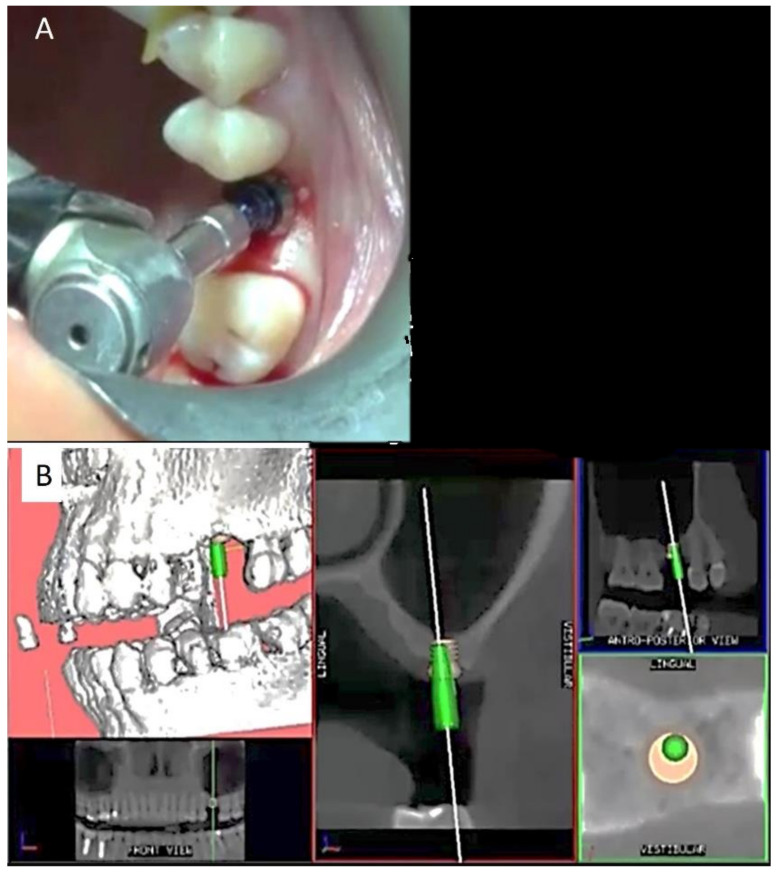
Clinical view of the implant screw sitting (**A**) and the screen view showing the final implant digital position, and the green pin representing the fixture superimposed on the 3D radiologic anatomy (**B**).

**Figure 5:**
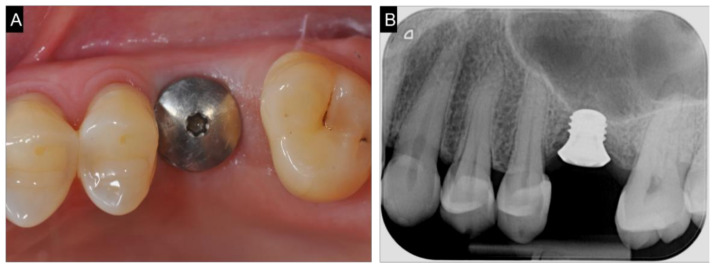
Clinical (**A**) and radiological (**B**) view after implant placement.

**Figure 6:**
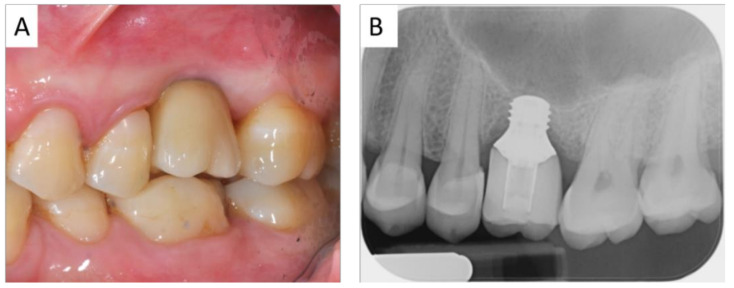
Clinical (**A**) and radiological (**B**) view after implant loading.

**Figure 7:**
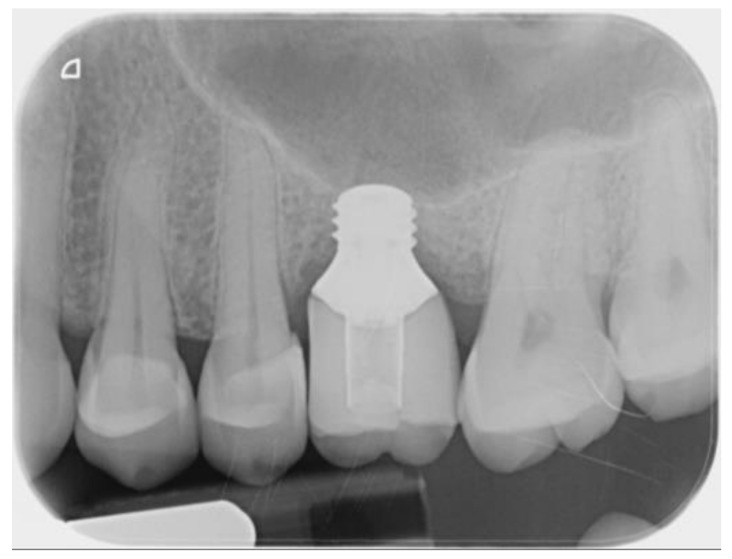
Radiological view at the end of follow-up.

After setting up the Navigation System under local anesthesia (mepivacaine with adrenaline 1:50,000) (Figure 2), a minimal crestal incision with no release was performed. One 4-mm-long and 4.1 mm wide SLActive^®^ soft tissue level implant was placed according to the site preparation instructions of the implant manufacturer (Institute Straumann AG, Basel, Switzerland); the implant achieved good primary stability (insertion torque ≥35 NCM). The wound was sutured with silk 4.0. See Figure 3, Figure 4, Figure 5, Figure 6 and Figure 7.

The patient was instructed to adhere to a soft diet for a few days and maintain appropriate oral hygiene with daily rinsing using a 0.2% chlorhexidine mouthwash. No complaints of post-operative discomfort, such as bleeding, edema, or pain, were reported in the immediate post-operative period, and no complications were recorded. After six weeks of healing, a straight conical abutment was connected, and impressions were taken with a customized tray. After one week, a provisional crown was delivered to the patient. The acrylic resin provisional crown was replaced with a definitive ceramic one after three months. Occlusal surfaces were modeled to reduce contacts in laterally and protrusion excursions, maintaining those in the maximum intercuspation position. General oral health instructions and professional oral hygiene every six months were recommended.

## 3. Results

The treatment objectives were achieved with a good occlusion, and the patient declared completely satisfaction. No surgical and prosthetic complications occurred up to three years follow up. The last endo-oral x-ray control did not evidence any variation in respect to the prosthetic loading time. No signs of mucositis were detected after a circumferential probing evaluation, with no bleeding on probing and a good mucosal sleeve around the implant shoulder.

## 4. Discussion

The hitherto most prolonged period of the investigation after short implant loading in posterior jaws is 15 years, with an overall 93.3% survival rate of ≤8.5 mm-long implants supporting cemented fixed partial dentures [4]. Up to 10-years follow-up, a cumulative 92.3% survival rate of 6/7 mm-long implants in mandibles [24] and a 91.7% survival rate for 6 mm-long implants in the maxilla were reported [5]. In contrast, no significant difference in terms of marginal bone resorption (MBR) and prosthetic complications between 10 mm and 6 mm-long implants was found [25]. Split mouth design RCTs comparing 5/6 mm-short implants with conventional ones after a sinus lift and mandibular inlay techniques recorded no statistically different results in terms of implant survival rate between the two groups after one [26,27], three [28], five years [29] and, only for posterior mandibles, eight years [30] and five years with the onlay technique [31]. Focusing on the posterior maxilla, Thoma et al. in a multicenter RCT on 137 implants observed a mean MBL of 0.54 mm ± 0.87 on short implants and 0.46 mm ± 1.00 on standard implants after sinus lift group, without differences in terms of prosthetic/biological complications, at five years post-loading, with a 98.5% survival rate in both groups [32]. Similar outcomes were reported by Nielsen et al. [33] and Gulijè et al. [34] up to three and five years post-loading, respectively.

In the last three years, almost ten systematic reviews were published on this topic, in addition of those with ≤1 years’ follow-up. On ≤6 mm short implants after a 1−5 years follow-up, survival rates ranging from 86.7% to 100%, with 94.1% weighted mean survival rate, 90% in the maxilla, and 96% in the mandible were reported [6,8]. In 2020, Rameh et al., limiting the inclusion criteria to a minimum of five-year follow-up, reported for 5/6 mm-long implants, an average of 95.54%, ranging between 86.7 and 98.5%, survival rate in the maxilla, and a 94.39%, ranging between 86.7 and 100%, in mandible [7]. Vazouras et al., including the studies on ≥4 and ≤6 mm ultra-short implants with a minimum follow-up of three years, calculated a 4% overall failure increasing to 10% after that [9]. The first reports on 4 mm ultra-short implants in posterior mandibles found a survival rate of 95.7% and 92.3%, after one and two years, respectively [35]. Two RCTs on 4 mm-long versus ≥10 mm-long implants in sinus lifts and inlay grafted mandibular sites, at four months [36] and one year follow up [10] confirmed similar results between the two groups, as did the study comparing 4 mm-short implants versus longer ones in GBR augmented posterior mandibles [11]. Terrassa et al. [37] and Leighton et al. [38] recorded a 100% survival rate both for 4 mm-long implants splinted to 8 mm-long implants in posterior maxilla after two years and for single crown supporting 4 mm-long implants in posterior mandible after three years of loading. The longest follow-up reported is five years, with 92.2% of survival in mandible [17].

All the studies convey that short implants could be a better choice considering the morbidity, the costs, and the duration of the reconstructive approaches. Nevertheless, a time-dependent higher risk of failure of ≤6 mm-long implants emerged, with a wide range of the survival rates, from 86.7% to 100%, and a calculated 24% higher risk for 1–5 years follow-up than with longer implants [9]. Regarding ≥5 years of loading of ≤5 mm-long screws, the literature is so far insufficient to recommend this approach [39].

Most of the studies did not find a positive correlation between the C/I ratio and the marginal bone resorption, without relevance of a single unit or a fixed splinting partial bridge as a prosthetic superstructure. Otherwise, the type of prosthesis seems to condition the integrity of the connection screws and the matching surfaces between the endo and extra-osseous structures [37]. In vitro studies have evidenced how occlusal loads were evenly distributed between the implants, leading to decreased stress concentration at the implant-abutment interface, implant neck, and surrounding bone [7,9,39,40]. A 9% failure rate of short implants restored with a single crown versus a 3% of splinted ones with bridges up to five years is associated with prosthetic more components’ damage [8,9]. Another critical factor conditioning the post-loading follow-up and the pre-loading period is the implant bed site preparation [7]. An early implant failure range of 1.49% [8], –7% [35], was reported, representing 42.9% of the total rate. The short implant placement in posterior jaws is not simple and requires some experience. A bone compressing procedure and, differently, a passive implant insertion is indicated for maxilla and mandible, respectively [41], since the bone quality, too cancellous or too cortical, can compromise the primary stability of the fixtures losing residual bone and mistaking the correct position. Slotte et al. stated that the bed preparation procedure must be done with great care to avoid over-drilling, according to careful planning and prosthetic construction design [35]. Specific correlations were found in vitro between 6 mm and 4 mm-short implants’ primary stability and implant bed preparation techniques [42,43].

Placing a mini fixture in a limited bone quantity in a specific location to avoid anatomical damage and a precise spatial orientation to respect the prosthetic demands can benefit from the DN technology. Thanks to DN support, the operator can follow in real-time the progression of surgical drills on the 3D radiological imaging while maintaining the sensory perception in the hands and can reduce or augment the pressure on the surgical instruments, deciding to underprepared or tapping the site; furthermore, in case of intra-operative errors, the digital system can be reprogrammed for an alternative plan. As the DN technology is adaptable to piezo-surgical tools different surgical instrumentations, the operator can further vary the implant bed preparation according to the specific clinical situation [19].

The implant positioning accuracy’s reliability using standard or tilted implants has been widely demonstrated as superior with than without the DN support [21,44,45]. Block et al. [46], using a second-generation navigation system, compared deviations using this system vs. the free-hand one. Three surgeons were involved and treated 100 partially edentulous patients. The results with navigation were [mean (SD)] 0.87 (0.42) mm at entry point, 1.56 (0.69) mm at the apex and 3.62° (2.73°) angular versus 1.15 (0.59) mm, 2.51 (0.86) mm and 7.69° (4.92°). No statistically significant differences were observed in navigated implant placement between individual clinicians. Stefanelli et al. [20], using DN technology, reported 136 implants inserted in 59 patients with the following deviations: 0.67 mm at coronal, 0.99 at apical, 0.55 at depth, and 2.5 as an angular error. Pellegrino et al., comparing the planned implant position with the real one with the DN support in the pre and post-operative CBCT, recorded a mean deviation of 1.19 ± 0.54 mm between the post-operative navigated implant position and the planned one. The mean error at the insertion point was 1.04 ± 0.47 mm, the mean error at the apical point was 1.35 ± 0.56 mm, the depth deviation was 0.43 ± 0.34 mm, and the axis deviation from the planned value was 6.46 ± 3.95 [22]. From these data, useful for short-implants seating appears the minimal deviation of the insertion depth, enabling the anatomic structures’ respect. Since the longer the implant, the more the error deviation from what pre-operatively planned, the application of this technology to short and ultra-short implants could take advantage of a major implant placement precision. Particularly relevant for the short-implants approach can be the parameter of the angular deviation. In this regard, it seems that a 4 mm-long implant can bear axial loads like a 10 mm-long one, no matter the prosthetic crown height, while an oblique load induces stresses up to three times stronger than the axial to the bone around and the abutment [23]. Regarding implant outcomes in terms of survival, the authors have reported a rate of 100% versus 93% in the navigated implantology versus a free-hand one, with a substantial reduction of the operative times [28,34]. In the reported case, only a 4 mm-ultra- short implant could be used. Primary stability and maintaining bone height on time appeared challenging to address, emphasizing the necessity of a precise implant bed preparation respecting the available bone height and considering the prosthetic loading. The surface properties of the fixtures used augmented the bone-to-implant contact surface and helped in obtaining primary stability immediately; the precision in implant positioning addressed the necessity not to confer any inclination to the implant to prevent it from any lateral force in consideration of the length on the prosthetic crown with a crown-implant ratio ≥2.5. The clinician who managed this case, who was not familiar with DN technology, needed minimal training to visualize the screen and the surgical field and was supported by an experienced operator. The digital static system option was not constrained by a drill-guiding template limiting the operator’s clinical skills and requiring more extended drills passing through the directional sleeves. This report may encourage one to verify the actual improvement of the digital navigation technology in support of ultra-short implant placement.

## 5. Conclusions

No clinical and radiographic variation was noted at three years follow-up after-loading with a single crown of a 4 mm-long implant placed in the maxilla with DN technology support. The accuracy of implant insertion could improve this mini-invasive approach’s reliability over time in these anatomic and prosthetic challenging situations.

## References

[1] Garaicoa-Pazmino C., del Amo F.S.L., Monje A., Catena A., Ortega-Oller I., Galindo-Moreno P., Wang H.-L. (2014). Influence of Crown/Implant Ratio on Marginal Bone Loss: A Systematic Review. J. Periodontol..

[2] Anitua E., Tapia R., Luzuriaga F., Orive G. (2010). Influence of implant length, diameter, and geometry on stress distribution: A finite element analysis. Int. J. Periodontics Restor. Dent..

[3] Baggi L., Cappelloni I., Di Girolamo M., Maceri F., Vairo G. (2008). The influence of implant diameter and length on stress distribution of osseointegrated implants related to crestal bone geometry: A three-dimensional finite element analysis. J. Prosthet. Dent..

[4] Anitua E., Alkhraisat M.H. (2019). 15-year follow-up of short dental implants placed in the partially edentulous patient: Mandible Vs maxilla. Ann. Anat. Anat. Anz..

[5] Rossi F., Lang N.P., Ricci E., Ferraioli L., Baldi N., Botticelli D. (2018). Long-term follow-up of single crowns supported by short, moderately rough implants—A prospective 10-year cohort study. Clin. Oral Implant. Res..

[6] Papaspyridakos P., De Souza A., Vazouras K., Gholami H., Pagni S., Weber H. (2018). Survival rates of short dental implants (≤6 mm) compared with implants longer than 6 mm in posterior jaw areas: A meta-analysis. Clin. Oral Implant. Res..

[7] Rameh S., Menhall A., Younes R. (2020). Key factors influencing short implant success. Oral Maxillofac. Surg..

[8] Ravidà A., Barootchi S., Askar H., Del Amo F.S.-L., Tavelli L., Wang H.-L. (2019). Long-Term Effectiveness of Extra-Short (≤6 mm) Dental Implants: A Systematic Review. Int. J. Oral Maxillofac. Implant..

[9] Vazouras K., De Souza A.B., Gholami H., Papaspyridakos P., Pagni S., Weber H.-P. (2019). Effect of time in function on the predictability of short dental implants (≤6 mm): A meta-analysis. J. Oral Rehabil..

[10] Bolle C., Felice P., Barausse C., Pistilli V., Trullenque-Eriksson A., Esposito M. (2018). 4 mm long vs longer implants in augmented bone in posterior atrophic jaws: 1-year post-loading results from a multicentre randomised controlled trial. Eur. J. Oral Implant..

[11] Rokn A.R., Monzavi A., Panjnoush M., Hashemi H.M., Kharazifard M.J., Bitaraf T. (2018). Comparing 4-mm dental implants to longer implants placed in augmented bones in the atrophic posterior mandibles: One-year results of a randomized controlled trial. Clin. Implant. Dent. Relat. Res..

[12] Malchiodi L., Ricciardi G., Salandini A., Caricasulo R., Cucchi A., Ghensi P. (2020). Influence of crown–implant ratio on implant success rate of ultra-short dental implants: Results of a 8- to 10-year retrospective study. Clin. Oral Investig..

[13] Lombardo G., Pighi J., Marincola M., Corrocher G., Simancas-Pallares M., Nocini P.F. (2017). Cumulative Success Rate of Short and Ultrashort Implants Supporting Single Crowns in the Posterior Maxilla: A 3-Year Retrospective Study. Int. J. Dent..

[14] Afrashtehfar K.I., Katsoulis J., Koka S., Igarashi K. (2021). Single versus splinted short implants at sinus augmented sites: A systematic review and meta-analysis. J. Stomatol. Oral Maxillofac. Surg..

[15] Lombardo G., Marincola M., Signoriello A., Corrocher G., Nocini P.F. (2020). Single-Crown, Short and Ultra-Short Implants, in Association with Simultaneous Internal Sinus Lift in the Atrophic Posterior Maxilla: A Three-Year Retrospective Study. Materials.

[16] Mangano F., Frezzato I., Frezzato A., Veronesi G., Mortellaro C., Mangano C. (2016). The Effect of Crown-to-Implant Ratio on the Clinical Performance of Extra-Short Locking-Taper Implants. J. Craniofac. Surg..

[17] Slotte C., Grønningsaeter A., Halmøy A.-M., Öhrnell L.-O., Mordenfeld A., Isaksson S., Johansson L.-Å. (2014). Four-Millimeter-Long Posterior-Mandible Implants: 5-Year Outcomes of a Prospective Multicenter Study. Clin. Implant. Dent. Relat. Res..

[18] Pellegrino G., Lizio G., Basile F., Stefanelli L.V., Marchetti C., Felice P. (2020). Dynamic Navigation for Zygomatic Implants: A Case Report about a Protocol with Intraoral Anchored Reference Tool and an Up-To-Date Review of the Available Protocols. Methods Protoc..

[19] Pellegrino G., Tarsitano A., Taraschi V., Vercellotti T., Marchetti C. (2018). Simplifying Zygomatic Implant Site Preparation Using Ultrasonic Navigation: A Technical Note. Int. J. Oral Maxillofac. Implant..

[20] Stefanelli L.V., DeGroot B.S., Lipton D., Mandelaris G. (2019). Accuracy of a Dynamic Dental Implant Navigation System in a Private Practice. Int. J. Oral Maxillofac. Implant..

[21] Pellegrino G., Bellini P., Cavallini P.F., Ferri A., Zacchino A., Taraschi V., Marchetti C., Consolo U. (2020). Dynamic Navigation in Dental Implantology: The Influence of Surgical Experience on Implant Placement Accuracy and Operating Time. An in Vitro Study. Int. J. Environ. Res. Public Health.

[22] Pellegrino G., Taraschi V., Andrea Z., Ferri A., Marchetti C. (2019). Dynamic navigation: A prospective clinical trial to evaluate the accuracy of implant placement. Int. J. Comput. Dent..

[23] Capatti R.S., Barboza M.S., Antunes A.N.D.G., Oliveira D.D., Seraidarian P.I. (2020). Viability of Maxillary Single Crowns Supported by 4-mm Short Implants: A Finite Element Study. Int. J. Oral Maxillofac. Implant..

[24] Friberg B., Gröndahl K., Lekholm U., Brånemark P.-I. (2000). Long-term Follow-up of Severely Atrophic Edentulous Mandibles Reconstructed with Short Branemark Implants. Clin. Implant. Dent. Relat. Res..

[25] Storelli S., Abbà A., Scanferla M., Botticelli D., Romeo E. (2018). 6 mm vs 10 mm-long implants in the rehabilitation of posterior jaws: A 10-year follow-up of a randomised controlled trial. Eur. J. Oral Implant..

[26] Pistilli R., Felice P., Cannizzaro G., Piatelli M., Corvino V., Barausse C., Buti J., Soardi E., Esposito M. (2013). Posterior atrophic jaws rehabilitated with prostheses supported by 6 mm long 4 mm wide implants or by longer implants in augmented bone. One-year post-loading results from a pilot randomised controlled trial. Eur. J. Oral Implant..

[27] Pistilli R., Felice P., Piattelli M., Gessaroli M., Soardi E., Barausse C., Buti J., Corvino V. (2013). Posterior atrophic jaws rehabilitated with prostheses supported by 5 × 5 mm implants with a novel nanostructured calcium-incorporated titanium surface or by longer implants in augmented bone. One-year results from a randomised controlled trial. Eur. J. Oral Implant..

[28] Esposito M., Pistilli R., Barausse C., Felice P. (2014). Three-year results from a randomised controlled trial comparing prostheses supported by 5-mm long implants or by longer implants in augmented bone in posterior atrophic edentulous jaws. Eur. J. Oral Implant..

[29] Felice P., Pistilli R., Barausse C., Piattelli M., Buti J., Esposito M. (2019). Posterior atrophic jaws rehabilitated with prostheses supported by 6-mm-long 4-mm-wide implants or by longer implants in augmented bone. Five-year post-loading results from a within-person randomised controlled trial. Int. J. Oral Implant..

[30] Felice P., Barausse C., Pistilli R., Ippolito D.R., Esposito M. (2018). Short implants versus longer implants in vertically augmented posterior mandibles: Result at 8 years after loading from a randomised controlled trial. Eur. J. Oral Implant..

[31] Pieri F., Forlivesi C., Caselli E., Corinaldesi G. (2017). Short implants (6 mm) vs. vertical bone augmentation and standard-length implants (≥9 mm) in atrophic posterior mandibles: A 5-year retrospective study. Int. J. Oral Maxillofac. Surg..

[32] Thoma D.S., Haas R., Sporniak-Tutak K., Garcia-Garcia A., Taylor T.D., Hämmerle C.H.F. (2018). Randomized controlled multicentre study comparing short dental implants (6 mm) versus longer dental implants (11–15 mm) in combination with sinus floor elevation procedures: 5-Year data. J. Clin. Periodontol..

[33] Nielsen H., Schou S., Isidor F., Christensen A.-E., Starch-Jensen T. (2019). Short implants (≤8 mm) compared to standard length implants (>8 mm) in conjunction with maxillary sinus floor augmentation: A systematic review and meta-analysis. Int. J. Oral Maxillofac. Surg..

[34] Guljé F.L., Raghoebar G.M., Vissink A., Meijer H.J.A. (2019). Single crowns in the resorbed posterior maxilla supported by either 11-mm implants combined with sinus floor elevation or 6-mm implants:A 5-year randomised controlled trial. Int. J. Oral Implantol..

[35] Slotte C., Grønningsaeter A., Halmøy A.-M., Öhrnell L.-O., Stroh G., Isaksson S., Johansson L.-Å., Mordenfeld A., Eklund J., Embring J. (2011). Four-Millimeter Implants Supporting Fixed Partial Dental Prostheses in the Severely Resorbed Posterior Mandible: Two-Year Results. Clin. Implant. Dent. Relat. Res..

[36] Esposito M., Zucchelli G., Barausse C., Pistilli R., Trullenque-Eriksson A., Felice P. (2016). Four mm-long versus longer implants in augmented bone in atrophic posterior jaws: 4-month post-loading results from a multicentre randomised controlled trial. Eur. J. Oral Implant..

[37] Torassa D., Naldini P., Calvo-Guirado J.L., Fernández-Bodereau E. (2020). Prospective, Clinical Pilot Study with Eleven 4-Mm Extra-Short Implants Splinted to Longer Implants for Posterior Maxilla Rehabilitation. J. Clin. Med..

[38] Leighton Y., Carpio L., Weber B., Dias F.J., Borie E. (2020). Clinical evaluation of single 4-mm implants in the posterior mandible: A 3-year follow-up pilot study. J. Prosthet. Dent..

[39] Lemos C.A.A., Ferro-Alves M.L., Okamoto R., Mendonça M.R., Pellizzer E.P. (2016). Short dental implants versus standard dental implants placed in the posterior jaws: A systematic review and meta-analysis. J. Dent..

[40] Toniollo M.B., Macedo A.P., Pupim D., Zaparolli D., de Mattos M.G.C. (2016). Three-Dimensional Finite Element Analysis Surface Stress Distribution on Regular and Short Morse Taper Implants Generated by Splinted and Nonsplinted Prostheses in the Rehabilitation of Various Bony Ridges. J. Craniofac. Surg..

[41] Renouard F., Nisand D. (2005). Short Implants in the Severely Resorbed Maxilla: A 2-Year Retrospective Clinical Study. Clin. Implant. Dent. Relat. Res..

[42] Calvo-Guirado J.L., Torres J.A.L., Dard M., Javed F., Martínez C.P.-A., De Val J.E.M.S. (2015). Evaluation of extrashort 4-mm implants in mandibular edentulous patients with reduced bone height in comparison with standard implants: A 12-month results. Clin. Oral Implant. Res..

[43] Kucukguven M.B., Topaloglu G., Isıkhan S.Y., Tosun E., Saysel M.Y. (2020). In Vitro Evaluation of the Primary Stability of Short Implants in Different Surgical Techniques. Int. J. Oral Maxillofac. Implant..

[44] Aydemir C.A., Arısan V. (2020). Accuracy of dental implant placement via dynamic navigation or the freehand method: A split-mouth randomized controlled clinical trial. Clin. Oral Implant. Res..

[45] Kramer F.-J., Baethge C., Swennen G., Rosahl S. (2004). Navigated vs. conventional implant insertion for maxillary single tooth replacement. Clin. Oral Implant. Res..

[46] Block M.S., Emery R.W., Lank K., Ryan J. (2017). Implant Placement Accuracy Using Dynamic Navigation. Int. J. Oral Maxillofac. Implant..

